# A Case of Fetus in Fetu

**Published:** 2012-06-01

**Authors:** Ghulam Mustafa, Bilal Mirza, Shahid Iqbal, Afzal Sheikh

**Affiliations:** Department of Pediatric Surgery, The Children's Hospital and the Institute of Child Health Lahore, Pakistan

**Keywords:** Fetus in fetu, Teratoma, Abdominal mass

## Abstract

Fetus in fetu is a rare developmental aberration, characterized by encasement of partially developed monozygotic, diamniotic, and monochorionic fetus into the normally developing host. A 4-month-old boy presented with abdominal mass. Radiological investigations gave the suspicion of fetus in fetu. At surgery a fetus enclosed in an amnion like membrane at upper retroperitoneal location was found and excised. The patient is doing well after the operation.

## INTRODUCTION

Fetus in fetu (FIF) is an uncommon pathology that results due to abnormal embryogenesis in a diamniotic monochorionic twin pregnancy with an incidence of 1 in 500000 births. The commonly accepted theory states that unequal division of blastocoele results in monozygotic, monochorionic, and diamniotic twins of unequal sizes following which the smaller twin encases into the normally developing twin; the mechanism of which is not known. This is followed by arrest of further growth of the encased fetus due to improper blood supply or inherent defects of the encased twin. Few authors consider FIF as an advanced form of teratoma [1-3]. We report another case of fetus in fetu diagnosed preoperatively with the help of radiological investigations.

## CASE REPORT

A 4-month-old male baby presented to our hospital with the complaint of palpable mass in the right hemi-abdomen noted by the parents one day back. The patient was born at full term with uneventful birth history. The baby achieved milestones normally. Abdominal examination revealed a non-tender mass with vague margins in the right hemi-abdomen. Laboratory investigations including alpha-fetoprotein were within normal limits. X-ray abdomen showed mass impression pushing the gut shadows to one side. Bones and calcifications were also evident in the right hemi-abdomen. Ultrasound of the abdomen revealed a heterogeneous mass with calcifications suggestive of teratoma. Abdominal CT scan showed a 9.2cm × 10.0cm heterogeneous mass containing fat, bones and soft tissues. The various bones were vertebrae, long bones like femur, tibia and fibula, and bones of hand/feet (Fig.1). Provisional diagnosis of FIF was made.

**Figure:**
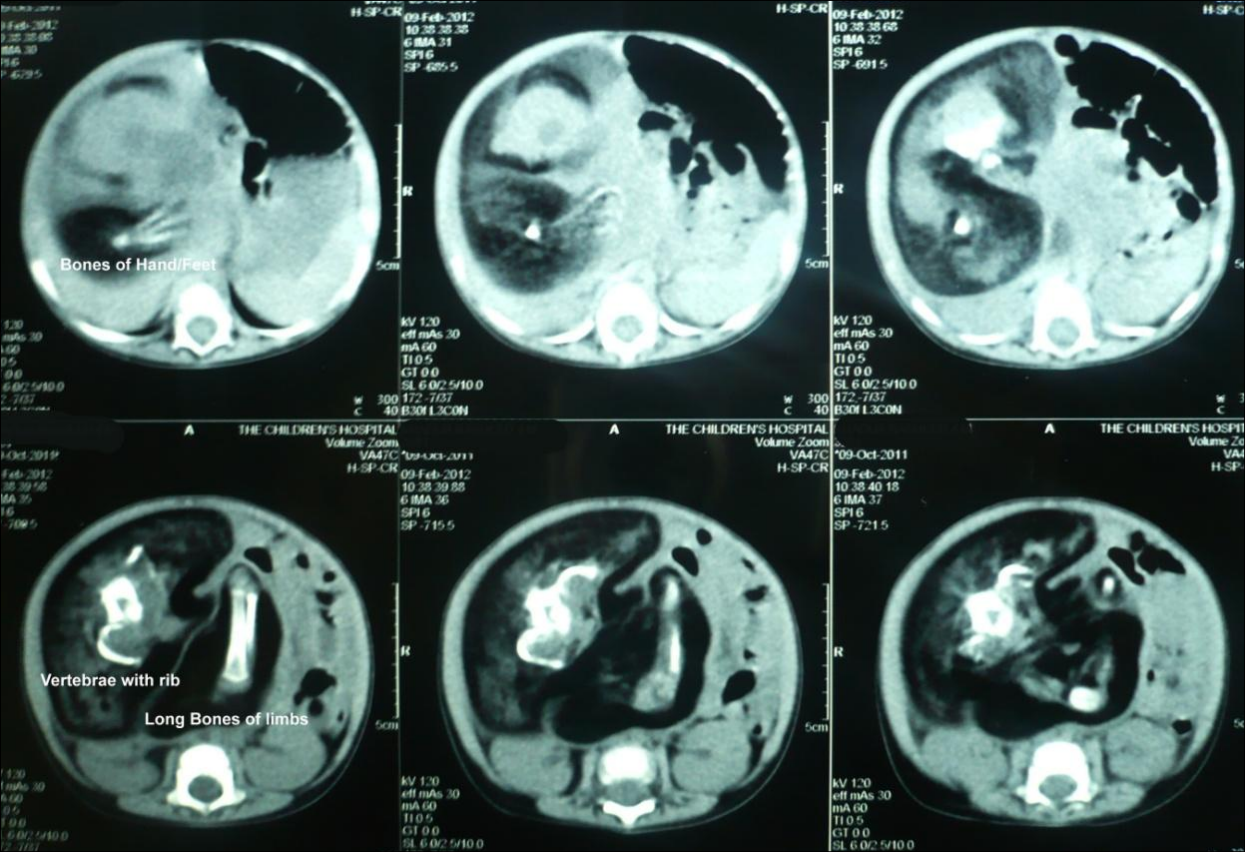
Figure 1: CT scan showing various kind of bones in FIF.

At operation, a mass covered in whitish-gray membrane, pushing the gut loops to the opposite side in the upper retroperitoneum, was found (Fig.2). The membrane was incised to find a fetiform mass floating in clear fluid having a few well differentiated and other rudimentary organs. The fetiform mass was suspended in the amnion like cavity with an umbilical cord like stalk (Fig.3). The mass with sac was mobilized and excised completely.

**Figure:**
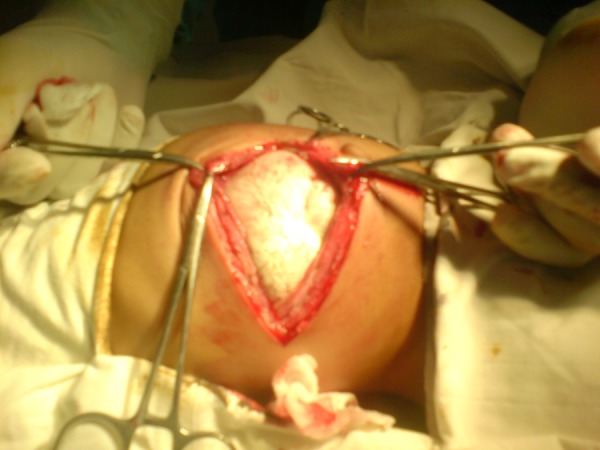
Figure 2: Amnion like covering of FIF.

**Figure:**
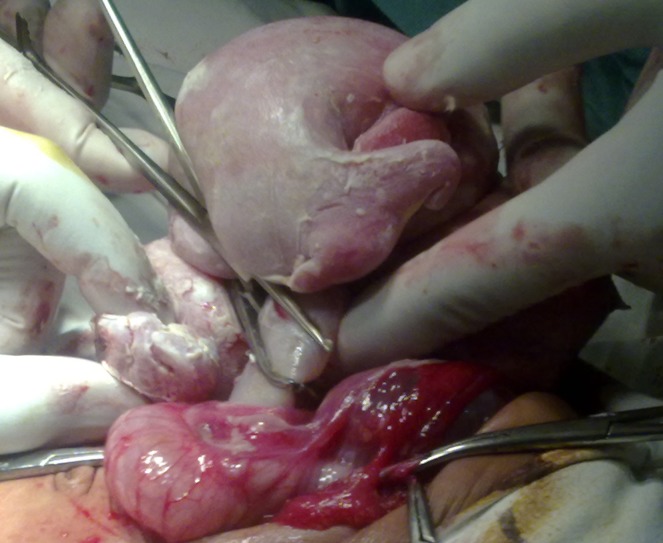
Figure 3: Umbilical cord like stalk- attachment of FIF.

Post operative recovery was uneventful. Patient was allowed orally on 3rd and discharged on 7th post operative day. The patient is currently being followed with alpha-fetoprotein and ultrasound abdomen. At six months follow up patient is doing well.

The FIF was of 13.75cm×12.5cm×6.25cm size with a weight of 500 grams. It was anencephalic having otic and nasal placodes and optic vesicles. The left upper limb was meromelic; lower limbs were sirenomelic. The umbilical cord had two vessels in it. The FIF had a scrotum like skin at the site of genitalia that lacked gonad in it. The FIF also lacked anus and genitalia (Fig.4). Plain radiography of the specimen revealed axial skeleton in the form of vertebrae, along with ribs, long bones of upper and lower limbs, and facial bones (Fig.5).

**Figure:**
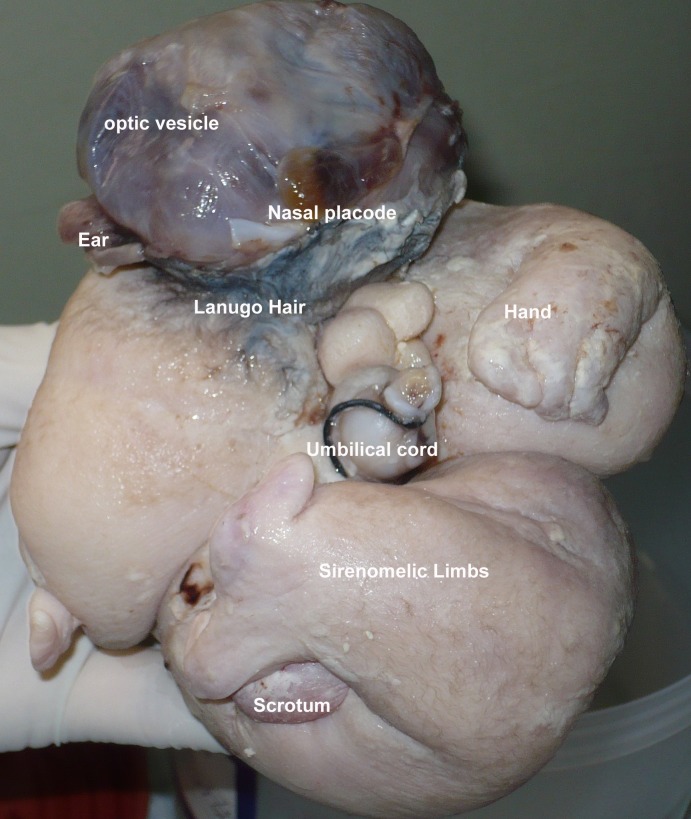
Figure 4: Various features of FIF.

**Figure:**
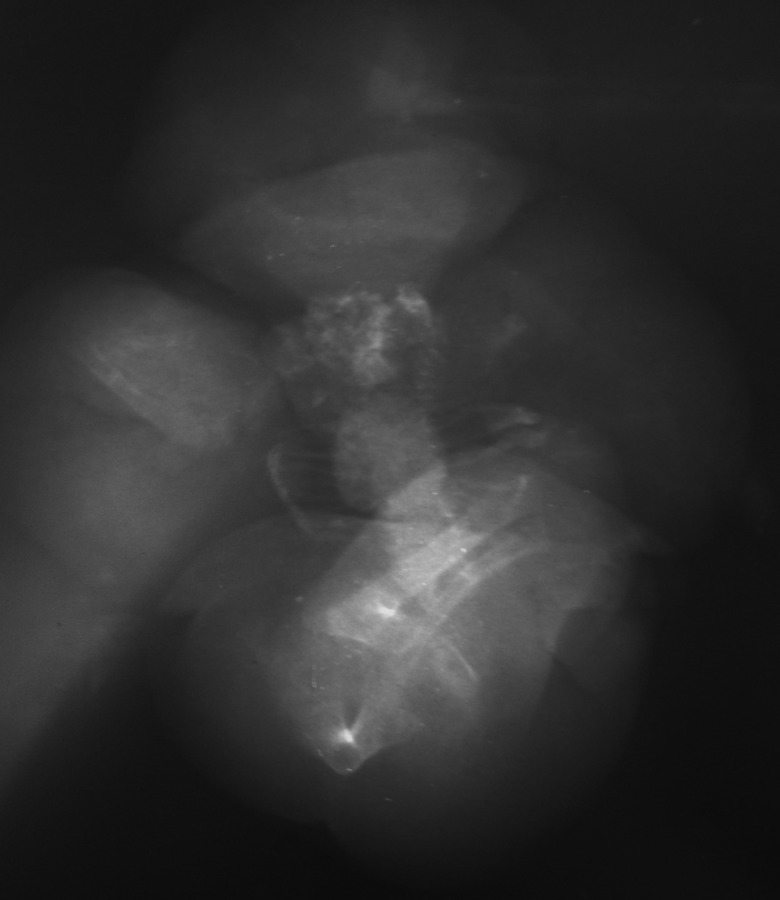
Figure 5: Radiograph of FIF showing ribs, vertebrae, hip bone, bones of face and limbs.

## DISCUSSION

The term Fetus in fetu was coined by Meckel during the late 18th century following which Willis described it as a rare condition where a malformed parasitic twin is found encased in the host especially in the retroperitoneal space. The other reported sites are abdomen, scrotum, cranium, kidneys, adrenals, mediastinum, and lymph nodes etc. FIF usually occurs as a single lesion however multiple FIF have also been reported, highest being 5. FIF is always a curiosity and to date about 200 cases have been reported in literature [3-6].

Most of the cases present during infancy, but late presentation has also been reported with the oldest patient presenting at 47-year. Male preponderance is noted in the reported cases. The major presenting complaint is a palpable abdominal mass, predominantly in upper abdomen. The other symptoms are secondary to the mass effect of the FIF such as, jaundice, hydronephrosis, intestinal obstruction, meconium peritonitis, respiratory distress, and vomiting [7-9].

Few reports describe antenatal diagnosis of FIF. Preoperative diagnosis can be made on plain radiographs and CT scan/MRI. The presence of vertebrae, long bones, bones of hands and feet etc are the common radiological findings. Visualization of a non-homogenous mass with bones especially vertebrae is considered pathognomonic of FIF. Failure to visualize vertebrae however does not rule out possibility of FIF. The other frequent differential is teratoma [1,5,10].

Most of the reported cases describe FIF suspended with an umbilical cord like stalk in an amnion like membrane containing fluid- equivalent to amniotic cavity. In few cases, the exact blood supply could be identified; in most of cases the blood supply was thought to come from the abdominal wall where amnion like membrane was in close approximation to it. Similarly, in our case the FIF was suspended in the fluid filled cavity with an umbilical cord like structure having two vessels in it. The FIF are usually anencephalic, with the vertebrae and limb-buds (long bones and bones of hands/feet can also present), and acardiac (rarely heart was found). In few cases vertebral column was not found however presence of mature enteric nervous plexi and melanocytes in the skin depicted the fetus would have passed the primitive streak stage of notochord development [1-5]. In our case the FIF was anencephalic, having primitive structures of nose, eyes and ears. One hand was well developed. The lower limbs were fused as in sirenomelia- long bones were palpable in the lower limbs.

Careful dissection of FIF should be done to avoid injury to the surrounding structures. A case of bile duct injury has been reported in literature. Complete excision of FIF along with covering membrane is necessary, as a case of malignant transformation of left over membrane is reported in literature. These cases are monitored with alpha-fetoprotein or beta-HCG, along with ultrasound and other radiological investigations [11,12]. We are following our patient on similar lines. 

## Footnotes

**Source of Support:** Nil

**Conflict of Interest:** None declared
